# Ion recombination correction factors ($P_{\text{ion}}$) for Varian TrueBeam high‐dose‐rate therapy beams

**DOI:** 10.1120/jacmp.v13i6.3803

**Published:** 2012-11-08

**Authors:** Stephen F. Kry, Richard Popple, Andrea Molineu, David S. Followill

**Affiliations:** ^1^ Department of Radiation Physics Radiological Physics Center The University of Texas M. D. Anderson Cancer Center Houston Texas USA; ^2^ Department of Radiation Oncology The University of Alabama at Birmingham Birmingham AL USA

**Keywords:** flattening filter free, FFF, $P_{\text{ion}}$, recombination, calibration, TG 51

## Abstract

Ion recombination is approximately corrected for in the Task Group 51 protocol by $P_{\text{ion}}$, which is calculated by a two‐voltage measurement. This measurement approach may be a poor estimate of the true recombination, particularly if $P_{\text{ion}}$ is large (greater than 1.05). Concern exists that $P_{\text{ion}}$ in high‐dose‐per‐pulse beams, such as flattening filter free (FFF) beams, may be unacceptably high, rendering the two‐voltage measurement technique inappropriate. Therefore, $P_{\text{ion}}$ was measured for flattened beams of 6, 10, 15, and 18 MV and for FFF beams of 6 and 10 MV. The values for the FFF beams were verified with 1/V versus 1/Q curves (Jaffé plots). $P_{\text{ion}}$ was also measured for electron beams of 6, 12, 16, 18, and 20 MeV on a traditional accelerator, as well as on the high‐dose‐rate Varian TrueBeam accelerator. The measurements were made at a range of depths and with PTW, NEL, and Exradin Farmer‐type chambers. Consistent with the increased dose per pulse, $P_{\text{ion}}$ was higher for FFF beams than for flattening filter beams. However, for all beams, measurement locations, and chambers examined, $P_{\text{ion}}$ never exceeded 1.018. Additionally, $P_{\text{ion}}$ was always within 0.3% of the recombination calculated from the Jaffé plots. We conclude that ion recombination can be adequately accounted for in high‐dose‐rate FFF beams using $P_{\text{ion}}$ determined with the standard two‐voltage technique.

PACS numbers: 87.56.‐v, 87.56.Da

## I. INTRODUCTION

The current standard for beam calibration in North America is the American Association of Physicists in Medicine Task Group (TG) 51 protocol.^(1)^ The most commonly used protocol internationally is the International Atomic Energy Agency's Technical Reports Series No. 398 protocol.^(2)^ A common feature of both protocols is the need to make corrections to the raw ionization readings to account for issues such as polarity effects, environmental conditions, electrometer calibration, and ion recombination.

The ion recombination correction factor, $P_{\text{ion}}$, is used to correct for incomplete signal collection associated with the recombination of ion pairs either along a given ionization track or between different ionization tracks. This correction has received theoretical attention^3^, ^7^ and has been studied for clinical radiotherapy scenarios.^6^, ^11^ Clinically, $P_{\text{ion}}$ is determined using the two‐voltage method^(12)^ (using high voltage [$V_{H}$] and low voltage [$V_{L}$] that produce measured ionization readings $M_{H}$ and $M_{L}$, respectively). For pulsed beams, $P_{\text{ion}}$ is described in TG 51 by:

(1) $$P_{ion}(V_{H})=\frac{1-\left(\frac{V_{H}}{V_{L}}\right)}{\left(\frac{M_{raw}^{H}}{M_{raw}^{L}}\right)-\left(\frac{V_{H}}{V_{L}}\right)}$$

Equation (1) is an approximation of the recombination effects that assumes a linear relationship between 1/M and 1/V. While this is reasonably accurate when there is little recombination,^1^, ^13^ the true relationship is known to be nonlinear,^(12)^ and the true recombination may be different than predicted with the two‐voltage technique. TG 51 recommends using the two‐voltage $P_{\text{ion}}$ value provided $P_{\text{ion}}<1.05$.^(1)^ In clinical practice, this condition is usually satisfied. However, the two‐voltage method has been shown to be notably inaccurate for both low electric field strength^(6)^ and very large doses per pulse.^14^, ^15^ Piermattei et al.,^(14)^ for example, showed substantial error in the recombination correction estimated by the two‐voltage technique for a high dose‐per‐pulse intraoperative electron therapy accelerator. That error corresponded to a 20% difference in dose to water measured by several ion chambers, as compared to a dose‐per‐pulse independent dosimeter.

Recently, new medical accelerators have been introduced that offer a high‐dose‐per‐pulse beam: the high‐dose‐rate flattening filter free (FFF) X‐ray modality. Because of the higher dose per pulse and correspondingly higher ionization density, concern has been raised that the standard two‐voltage method for ion recombination correction may be inappropriate. This could correspondingly introduce inaccuracies into the calibration of photon and electron beams. Therefore, in this study we measured $P_{\text{ion}}$ for traditional flattened beams, as well as high‐dose‐rate FFF beams with a variety of Farmer‐type chambers to determine if the existing two‐voltage method is appropriate for FFF beams. The validity of these measurements was confirmed with 1/Q versus 1/V Jaffé plots.

## II. MATERIALS AND METHODS

Measurements of $P_{\text{ion}}$ were made using the two‐voltage method outlined in the TG 51 protocol with voltages of ‐300 V and ‐1 50 V. After any change in voltage (at this step and for all other instances where the voltage was changed), ion chamber readings were discarded until a stable signal was measured over multiple readings (i.e., the readings did not change monotonically and no readings differed by more than 0.15%). At least three such nontrending readings of 200 MU each were collected at each voltage for each measurement of $P_{\text{ion}}$. Measurements were made inside a large water phantom at a source‐to‐surface distance of 100 cm. The field size was $10\times 10{\;\text{cm}}^{2}$ for photon beams, or the reference cone for electron beams.

First, $P_{\text{ion}}$ was measured for conventional flattened photon beams and associated electron beams with one of two Exradin model A‐12 cylindrical Farmer ion chambers (Standard Imaging, Middleton, WI). These measurements were made on several Varian 21EX linear accelerators (Varian Medical Systems, Inc., Palo Alto, CA). $P_{\text{ion}}$ was measured for photon beam energies of 6, 10, 15, and 18 MV at a depth of 10 cm, and for electron beam energies of 6, 12, 16, 18, and 20 MeV at a depth of $d_{\text{ref}}$. The low‐energy photon beams (6 and 10 MV) have a dose per pulse at $d_{\text{max}}$ of 0.03 cGy/pulse, while the high‐energy photon beams have a dose per pulse at $d_{\text{max}}$ of 0.056 cGy/pulse; the electron beams all have a dose per pulse at $d_{\text{max}}$ of 0.083 cGy/pulse (Varian Medical Systems, personal communiqué).

Next, $P_{\text{ion}}$ was measured on a Varian TrueBeam accelerator (Varian Medical Systems), which has traditional flattened beams, but also offers flattening filter free (high‐dose‐rate) photon beams that have a high dose per pulse. $P_{\text{ion}}$ was measured for the 6 MV and 10 MV high‐dose‐rate FFF beams. The dose per pulse at $d_{\text{max}}$ is 0.08 cGy/pulse and 0.13 cGy/pulse for the 6 MV and 10 MV FFF beams, respectively (Varian Medical Systems, personal communiqué). While measurements were made at the maximum nominal dose rate for each energy, the dose rate on the TrueBeam accelerator is reduced by dropping pulses (Varian Medical Systems, personal communiqué), meaning that the dose per pulse (and therefore the recombination and $P_{\text{ion}}$ correction) is independent of the nominal dose rate. For each of these FFF beams, $P_{\text{ion}}$ was measured at a depth of 10 cm and at $d_{\text{max}}$. Measurements of $P_{\text{ion}}$ were made with a PTW TN30013 (PTW, Freiburg, Germany), an NEL 2571 (QADOS, Berkshire, UK), and an Exradin A‐12 Farmer‐type ion chamber (Standard Imaging Inc.).

Finally, $P_{\text{ion}}$ was also measured for the high dose rate TrueBeam electron beams (6, 9, 12, 15, and 18 MeV). The $P_{\text{ion}}$ correction factor was measured with the Exradin A‐12 ion chamber at two depths for each electron beam: $d_{\text{ref}}$ and $R_{50}$.

To verify the $P_{\text{ion}}$ values, the inverse of the collected charge (1/Q) was plotted versus the inverse of the applied voltage (1/V) for the 6 MV and 10 MV FFF beams (i.e., Jaffé‐plots). The collected charge from 200 MU was measured as a function of chamber voltage, which was varied between 100 and 400 V. Measurements were conducted at $d_{\text{max}}$ for the Exradin, PTW, and NEL Farmer‐type ion chambers. The measured signal was extrapolated to 1/1/V=0 (infinite voltage) to estimate the recombination effects at 300 V. These Jaffé;‐plot–based recombination factors were compared to the $P_{\text{ion}}$ values determined with the two‐voltage method.

## III. RESULTS

For the conventional flattened photon beams examined in this study, the mean and range of the measured $P_{\text{ion}}$ values are presented in Table 1 for the 6, 10, 15, and 18 MV X‐ray beams. The results show very consistent values of $P_{\text{ion}}$ for the Exradin chambers, varying by no more than 0.2% over the 6–17 measured beams for each energy. Correspondingly, the standard deviations of the $P_{\text{ion}}$ values for each energy were also small, being only 0.0005 on average. The consistency of these results indicates that this measurement approach was very consistent, that there was little construction difference between the two ion chambers, and also that the dose per pulse generated by the different accelerators (which were operated and maintained at different facilities) nevertheless all generated very consistent doses per pulse. The value of $P_{\text{ion}}$ increased as the dose per pulse at 10 cm depth increased (that is, $P_{\text{ion}}$ was smallest for the 6 MV beam and largest for the 18 MV beam). Consistent with clinical experience, all $P_{\text{ion}}$ values were much less than 1.05.

**Table 1:** $P_{\text{ion}}$ values (at 300 V) for conventional flattened photon beams of different energies measured on several Varian 21 EX accelerators. The mean $P_{\text{ion}}$ and the maximum and minimum of the measurements are shown. Measurements were taken at a depth of 10 cm with an Exradin A‐12 chamber.

*Parameter*	*Energy*			
	*6 MV*	*10 MV*	*15 MV*	*18 MV*
No. of accelerators	17	16	7	7
Mean $P_{\text{ion}}$	1.003	1.003	1.005	1.006
Maximum $P_{\text{ion}}$	1.004	1.004	1.006	1.006
Minimum $P_{\text{ion}}$	1.002	1.002	1.004	1.006

Similar data are shown in Table 2, including the mean and range of $P_{\text{ion}}$ values measured for the 4–30 electron beams at each energy. The values of $P_{\text{ion}}$ were notably higher for the electron beams than they were for the photon beams (maximum of 1.017) because of the larger dose per pulse from these beams. The values of $P_{\text{ion}}$ were also consistent across electron energies, reflecting the dose per pulse being independent of electron beam energy. The range of measured values of $P_{\text{ion}}$ at each energy was slightly larger (0.4%–0.5%) for electron beams than was seen for photon beams. This indicates that either electron beams (in terms of dose per pulse) are somewhat less consistent across accelerators than photon beams, or that there is greater uncertainty in dose measurements with electron beams. As with the photon beams, all values of $P_{\text{ion}}$ were much less than 1.05.

**Table 2:** Measured $P_{\text{ion}}$ values (at 300 V) for conventional electron beams at $d_{\text{ref}}$ in water measured on several accelerators with an Exradin A‐12 chamber.

	*Energy*				
	*6 MeV*	*12 MeV*	*16 MeV*	*18 MeV*	*20 MeV*
No. of accelerators	30	26	4	7	19
Mean $P_{\text{ion}}$	1.013	1.013	1.015	1.012	1.014
Maximum $P_{\text{ion}}$	1.015	1.016	1.017	1.015	1.015
Minimum $P_{\text{ion}}$	1.010	1.011	1.013	1.010	1.010

Table 3 shows the values of $P_{\text{ion}}$ for the FFF beams at a depth of 10 cm and at $d_{\text{max}}$. The values for $P_{\text{ion}}$ were higher for the FFF beams than the values for the flattening filter beams of equivalent nominal energy and depth noted in Table 1. At 6 MV, $P_{\text{ion}}$ was higher for the FFF beam by 0.3%, whereas it was 0.5% higher for the 10 MV FFF beam. $P_{\text{ion}}$ values were higher at $d_{\text{max}}$ than at a depth of 10 cm because of the increased dose per pulse at that location. At 6 MV, the average recombination at 10 cm depth (1.0063) was 64% of the recombination at $d_{\text{max}}$ (1.010), which is consistent with the relative dose rate between these locations: the nominal ${\text{PDD}}_{10}$ for this beam is 64% (Varian Medical Systems, personal communiqué). At 10 MV, the recombination at 10 cm depth was 80% of the recombination at $d_{\text{max}}$ (1.012 versus 1.015), which compares reasonably well with the difference in dose rate: the nominal ${\text{PDD}}_{10}$ for this beam is 72% (Varian Medical Systems, personal communiqué).

**Table 3:** Measured $P_{\text{ion}}$ values at 300 V for FFF beams at a depth of 10 cm in water and at $d_{\text{max}}$ with three ion chambers.

*Ion Chamber*	*6 MV FFF*		*10 MV FFF*	
	*10 cm*	$d_{\text{max}}$	*10 cm*	$d_{\text{max}}$
Exradin A‐12	1.006	1.009	1.010	1.014
PTW TN30013	1.005	1.008	1.011	1.013
NEL 2571	1.008	1.013	1.015	1.018

The three ion chamber models generated $P_{\text{ion}}$ values that varied by as much as 0.5%. This difference is not a random variation, but rather represents different mechanical structures between the different models of ion chamber — particularly differences in electrode separation. Previous studies have also found that different chambers have different recombination rates.^(16)^

Measured $P_{\text{ion}}$ values for the high‐dose‐rate electron beams from the TrueBeam accelerator are presented in Table 4. All values were less than 1.012 and were slightly smaller than $P_{\text{ion}}$ values measured for lower‐dose‐rate electron beams (Table 2). The $P_{\text{ion}}$ value was independent of energy, but did increase from $R_{50}$ to $d_{\text{ref}}$. The recombination at $d_{\text{ref}}$ was almost, but not quite, double the recombination at $R_{50}$. Similarly, the dose rate is very nearly double at $d_{\text{ref}}$ as compared to $R_{50}$. This indicates that most of the recombination is volume recombination (occurring between different ionization tracks), which should scale with dose rate. The recombination not quite doubling as the dose rate doubles is an indication that there is some initial recombination as well (occurring along a given track).^(16)^

**Table 4:** Measured $P_{\text{ion}}$ values at 300 V for high‐dose‐rate (1000 MU/min) electron beams at two depths in water using an Exradin A‐12 chamber.

*Depth*	*Energy*				
	*6 MeV*	*9 MeV*	*12 MeV*	*15 MeV*	*18 MeV*
$d_{\text{ref}}$	1.011	1.011	1.012	1.011	1.011
$R_{50}$	1.007	1.007	1.006	1.007	1.007

**Figure 1:**
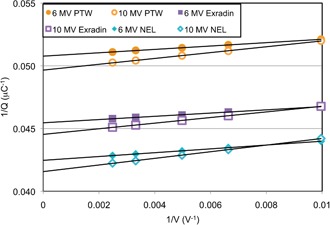
The inverse of the collected charge versus the inverse of the applied voltage for the 6 MV FFF beam and the 10 MV FFF beam for three ion chambers examined in this study (PTW, Exradin, and NEL). A linear best‐fit line through the data is included for each series.

To verify the validity of the $P_{\text{ion}}$ values measured above, the 1/Q versus 1/V data were plotted (Fig. 1). Different chambers showed different intercepts because of the difference in chamber volume, and different energies showed different slopes because of different dose rates. Nevertheless, for each energy and chamber, the measured signal showed a very linear relationship with applied voltage, as indicated by the linear best‐fit lines shown on the graph. Each best‐fit line was extrapolated back to 1/1/V=0 to determine the value of Q at infinite potential (no recombination). The ratio of this signal to the signal at 300 V was considered the Jaffé;‐plot–based recombination, which was compared to recombination based on the two‐voltage technique in Table 5. This table shows that the two‐voltage technique was accurate compared to the Jaffé‐plot approach within 0.3% at worst, and was usually accurate within 0.1%–0.2%. For comparison, the uncertainty in the Jaffé‐plot–based factor was estimated by conducting a linear regression of the data (in SPSS v.18), which showed an uncertainty in the intercept (Q at infinite potential) of less than 0.01% for all data series. This, when combined with the uncertainty in the measurement at 300 V (<0.15% per the reproducibility of the measurements), results in a final uncertainty in the Jaffé‐plot‐based recombination factor of less than 0.15%. Therefore, there is some small difference between the Jaffé‐plot–based correction factor and the two‐voltage technique‐based correction factor. However, the accuracy of the two‐voltage technique is sufficient for clinical practice, and is consistent with the accuracy of the two‐voltage technique stated by the TG 51 protocol for traditional flattened beams.

**Table 5:** Recombination factors at 300 V based on the two‐voltage technique ($P_{\text{ion}}$), and based on a Jaffé‐plot (1/V versus 1/Q curve).

*Ion Chamber*	*6 MV FFF*		*10 MV FFF*	
	$P_{ion}$	*Jaffé‐plot*	$P_{ion}$	*Jaffé‐plot*
Exradin A‐12	1.009	1.009	1.014	1.017
PTW TN30013	1.008	1.008	1.013	1.015
NEL 2571	1.013	1.011	1.018	1.020

## IV. DISCUSSION

The ion recombination correction factor, $P_{\text{ion}}$, was determined for three clinical ion chambers under high‐dose‐per‐pulse conditions that can be encountered with modern FFF beams. For all beams and ion chambers examined, recombination was well described with the two‐voltage $\left(P_{\mathrm{ion}}\right)$ technique. Even for the largest recombination measured, this approach agreed within 0.3% (and usually within 0.1%–0.2%) with Jaffé‐plot results. Correspondingly, we conclude that the standard two‐voltage method (Eq. 1) is suitable for all the measurement conditions we examined.

The uncertainty introduced by the two‐voltage approximation (~ 0.15%) appears to be the dominant source of uncertainty when accounting for recombination. The uncertainty in the ion chamber readings (the spread between the three readings that comprised each measurement point) was typically < 0.1%. Similarly, for photon beams, the spread between different setups and different machines was only ±0.1% (Table 1), indicating that different setups were not a large contributor to the uncertainty in $P_{\text{ion}}$.

**Figure 2:**
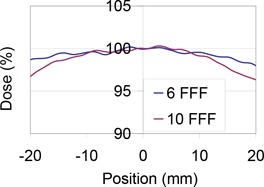
Dose profiles over the central 4 cm of large 6 MV FFF and 10 MV FFF fields. Decreased dose away from the central axis is characteristic of FFF beams.

In addition to the above uncertainty involved in determining $P_{\text{ion}}$ in any beam, FFF beams also have additional uncertainty because of potential partial volume effects. Specifically, the peaked radiation field will result in partial volume averaging effects in large‐volume Farmer‐type chambers. The magnitude of this error was evaluated by comparing film profiles of FFF beams (6 MV and 10 MV) to the size of Farmer‐type ion chambers (approximately 2 cm in length). Figure 2 shows a close‐up of the central 4 cm of the measured beam profiles. For both the 6 MV FFF and 10 MV FFF beams, the profile is relatively flat over the central 2 cm, and is comparably flat for both energies over this range. Nevertheless, averaging the signal over the size of a Farmer‐type ion chamber would correspond to an underestimation of the true central axis dose by 0.2% for both the 6 MV and 10 MV beams. Such a correction could be readily made to the calibration of the photon beams. However, the error introduced by ignoring the partial volume effect is small, compared to other errors in beam calibration (such as setup errors, or errors in $P_{\text{ion}}$ based on the two‐voltage technique).

Our study focused on the ion recombination correction factor as it pertains to reference beam calibration. Recombination may also be relevant for relative ion chamber measurements, such as percent depth dose curves. For such measurements, $P_{\text{ion}}$ is usually neglected based on the assumption that it is sufficiently constant over the range of measurement conditions (e.g., constant with depth). For the FFF beams, some variation in $P_{\text{ion}}$ was observed — for example, between a depth of 10 cm and $d_{\text{max}}$, the NEL chamber showed up to a 0.5% change in recombination in the 6 MV FFF beam. For precise dosimetry, this change in recombination may need to be considered.

**Figure 3:**
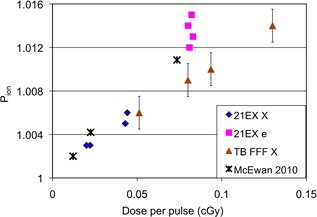
Measured $P_{\text{ion}}$ versus dose per pulse for X‐ray beams of 21 EX accelerators (“21EX X” from Table 1), electron beams of 21 EX accelerators (“21EX e” from Table 2), FFF X‐ray beams from a Varian TrueBeam accelerator (“TB FFF X” from Table 3), and from McEwan.^(16)^ Error bars on the FFF TrueBeam data are based on the accuracy of the two‐voltage technique established in Table 5 (0.15%). Doses per pulse at measurement locations not at $d_{\text{max}}$ (e.g. 10 cm depth or $d_{\text{ref}}$) were determined from the dose per pulse at $d_{\text{max}}$ and PDDs from either Varian golden beam data or clinical data.

The results in this study are supported by recent work by McEwen,^(16)^ who reported recombination factors for different chambers as a function of dose per pulse. McEwen's data for an Exradin A‐12 chamber are shown in Fig. 3, along with data from the current study using the Exradin A‐12 chamber. Electron beams from the TrueBeam are excluded because the manufacturer was unable to confirm the dose per pulse for these beams; however, if the dose per pulse is consistent with that of the 21 EX accelerator, the recombination is consistent with the rest of the data. Figure 3 shows that the recombination factors found in the current study are consistent with those found by McEwen. It also shows that the recombination in the high‐dose‐per‐pulse FFF photon beams is consistent with the recombination in the traditional 21 EX photon beams: increasing linearly with dose per pulse. While the electron beams showed greater variability in recombination than the photon beams, this difference was small, particularly in the context of clinical calibration.

## V. CONCLUSIONS

Even for the greatest dose per pulse evaluated in this study, based on the Varian TrueBeam accelerator high‐dose‐rate FFF photon beams, $P_{\text{ion}}$ was found to accurately account for ion recombination. The two‐voltage method used by the TG 51 calibration protocol^(1)^ can therefore be used to estimate the ion recombination correction factor for FFF beam calibration.
